# Analysis of prognostic factors for Tis-2N0M0 early glottic cancer with different treatment methods

**DOI:** 10.1016/j.bjorl.2020.06.013

**Published:** 2020-08-06

**Authors:** Guanyu Wang, Guodong Li, Jianjun Wu, Penghui Song

**Affiliations:** a Heping Hospital Affiliated to Changzhi Medical College, Department of Radiotherapy, Changzhi, China; b Shanxi Provincial People’s Hospital Affiliated to Shanxi Medical University, Department of Otolaryngology, Taiyuan, China

**Keywords:** Glottic cancer, 5-year survival rate, Laser microsurgery, Radiotherapy, Prognostic factors

## Abstract

**Introduction:**

In many regions, laryngeal carcinoma is a common upper respiratory tract cancer, most commonly involving the glottic region. The treatment of early glottic cancer includes radiotherapy, open surgery and laryngeal laser microsurgery. However, the preferred treatment for early glottic cancer is still controversial.

**Objectives:**

To study the factors affecting the 5-year survival rate of T_is-2_N_0_M_0_ early glottis cancer and to demonstrate the oncological safety of different treatments.

**Methods:**

144 patients with early glottic cancer were analyzed retrospectively. All patients were clinically node negative. 53 patients underwent open surgery, transoral CO_2_ laser microsurgery in 46 cases and radiotherapy in 45 cases. The patients were followed up for 26 − 84 months, with an average follow-up period of 62.9 months.

**Results:**

The 5-year overall survival was 82.6%. The 5 year survival rates of open surgery, laser microsurgery and radiotherapy were 83.0%, 82.6% and 82.2%, respectively. There was no significant difference in 5-year survival rate among the three treatments (*p* =  0.987). In multivariate analysis, age, T-stage, pathological grading, and anterior commissure involvement were important prognostic factors for early glottic cancer.

**Conclusion:**

There was no significant difference in 5 year survival rate among patients treated by either radiotherapy, laser microsurgery or open surgery for early glottic cancer. We urge more attention to the age, T-stage, pathological grade, and anterior commissure involvement of the patients.

## Introduction

In many regions, laryngeal carcinoma is a common upper respiratory tract cancer and most commonly involves the glottic region. Early glottic cancer is a highly curable disease. The 5-year local control rate in early glottic cancer patients is in the range of 85%–95% for T_1_N_0_M_0_ and in the range of 60%–80% for T_2_N_0_M_0_.^1^, ^2^, ^3^, ^4^, ^5^ The treatment of early glottic cancer includes radiotherapy, open surgery and laryngeal laser microsurgery. Laser microsurgery and radiotherapy have undergone significant advancement in the past few years leading to a decrease in open partial laryngectomy. However, the preferred treatment for early glottic cancer is still controversial. Laser microsurgery requires only a short hospital stay, but radiotherapy can maintain an optimal voice quality. Many studies have shown that voice preservation is better following radiotherapy than that after any surgical operation.^6^, ^7^, ^8^, ^9^ But there are also studies that do not support this conclusion.^10^ Therefore, the choice of treatment for patients with early glottic cancer should take into account the patient’s occupation, treatment cost, concomitant disease, length of stay and, most importantly, the patient’s preference.

The purpose of this study was to review the factors affecting the 5-year survival rate of T_is-2_N_0_M_0_ early glottis cancer and to prove the oncological safety of different treatments. In our hospital, our methods for the treatment of early glottic cancer include radiotherapy, laryngeal laser microsurgery and open surgery. In this study, we tried to assess prognostic factors, including gender, age, treatment, T-stage, pathological grade and anterior commissure involvement.

## Methods

### Patients

144 patients with early glottic cancer were analyzed retrospectively. These patients had complete data and follow-up records. In this study, the stage of tumor was determined on the basis of the clinical findings and classified according to the criteria of the Union for International Cancer Control (UICC). All patients were clinically node-negative. The tumor status was evaluated by routine Computerized Tomography (CT)/Magnetic Resonance Imaging (MRI) and fiberoptic laryngoscopy under local anesthesia.

### Treatments

The study included 132 males (91.7%) and 12 females (8.3%), aged 39 to 83 years, with a mean age of 52.4 years. A preoperative CT or MRI was performed in all patients to rule out any infiltration of the cartilaginous framework. 53 patients underwent open surgery (including total laryngectomy in 4 cases, partial laryngectomy in 49 cases), transoral CO_2_ laser microsurgery in 46 cases and radiotherapy in 45 cases (Table 1). No patient underwent prophylactic neck dissection. 45 patients received radiotherapy. The total dose was 66 Gy, 2.0 Gy per day for 33 treatments. The course of treatment was 7 weeks. During the treatment, radiation oncologists and otolaryngologists evaluated tumor regression by laryngoscopic examination. Each patient was examined once a month in the first year, every 3 months from the second to the third year, and every 6 months thereafter. Fiberoptic laryngoscopy was performed at each revisit. When follow-up laryngoscopy detected a suspicious lesion, biopsy specimens were examined to rule out tumor recurrence. The patients were followed up for 26 − 84 months, with an average follow-up period of 62.9 months. The diagnosis of local recurrence includes clinical examination, pathological diagnosis and fiberoptic laryngoscopy under local anesthesia.

**Table 1:** Distributions of GC patients according to the UICC 2002 staging system (n).

	T_is_	T_1_	T_2_	Total
Radiotherapy	0	31	14	45
Open surgery	0	15	38	53
Laser microsurgery	6	31	9	46
Total	6	77	61	144

### Statement of ethics

All experiments have been approved by the ethics committee of Heping Hospital Affiliated to Changzhi Medical College (nº 2019006) and conform to the guidelines of the local ethics committee.

### Statistical analysis

All statistical analyses were performed using the SPSS 25 software. Overall survival was calculated using the Kaplan-Meier curves. Predictive factors of survival were identified by univariate and multivariate analysis considering the following variables: gender, age (≥ 60 years vs. < 60 years), treatment, T-stage, pathological grading, and involvement of the anterior commissure (yes vs. no). For multivariate analysis, all variables that were statistically significant in the log-rank tests were included in the Cox proportional hazards models.^11^ A *p*-value of 0.05 or less was considered to be statistically significant.

## Results

**Figure 1:**
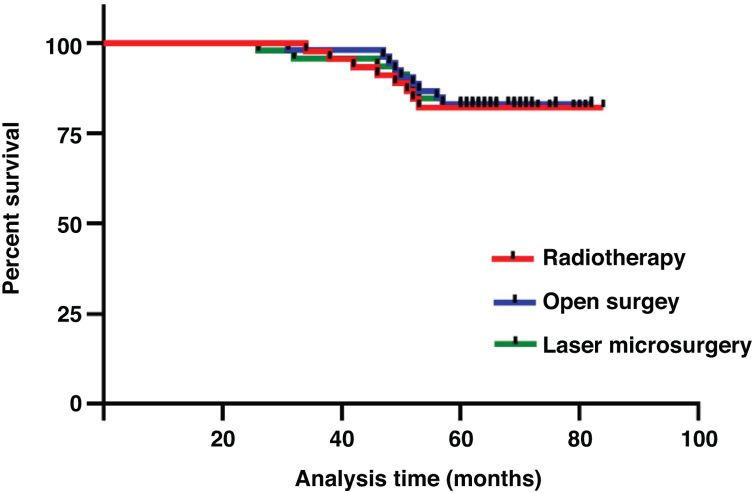
Comparison of 5-year survival rate of three treatments (*p* =  0.987).

The three treatment protocols of the patients are summarized in Table 1. The 5-year overall survival was 82.6%. The 5-year survival rates of open surgery, laser microsurgery and radiotherapy were 83.0%, 82.6% and 82.2%, respectively. There was no significant difference in 5-year survival rate among the three treatments (χ^2^ = 0.026, *p*  = 0.987) (Fig.1). A total of 25 patients died, of which 5 cases were related to laryngeal cancer, and the rest were due to secondary cancers or to intercurrent disease.

**Figure 2:**
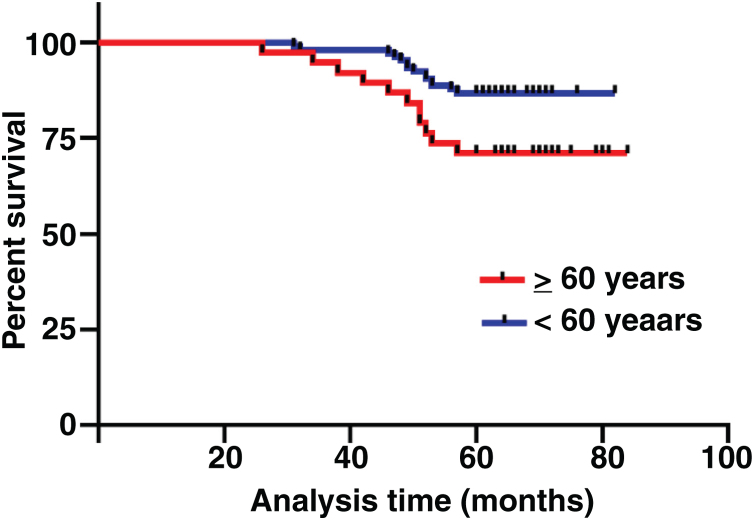
Comparison of 5-year survival rate by age group (*p* =  0.022).

**Figure 3:**
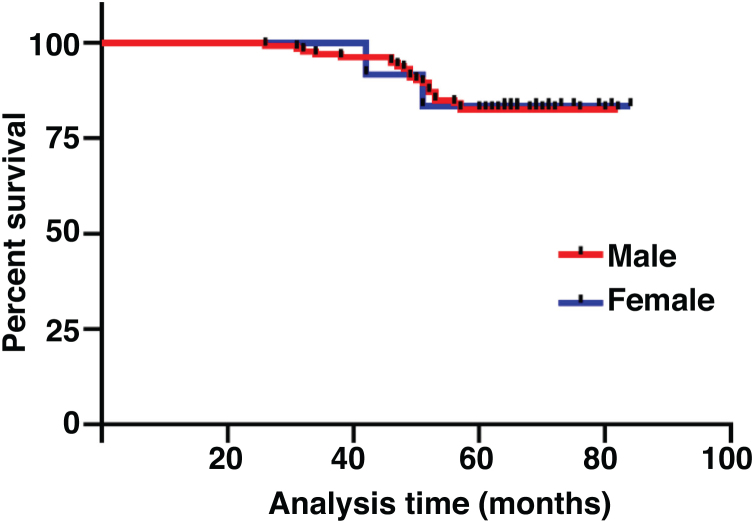
Comparison of 5-year survival rates by gender (*p* =  0.970).

**Figure 4:**
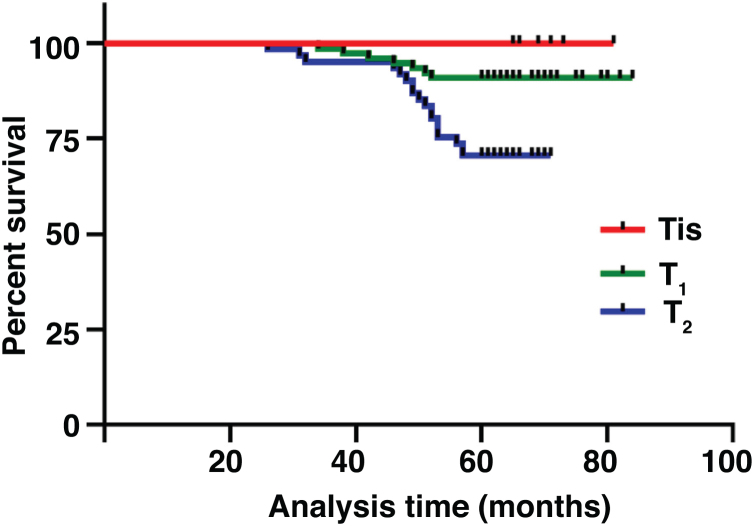
Comparison of 5-year survival rate according to T-stage (*p* =  0.005).

**Figure 5:**
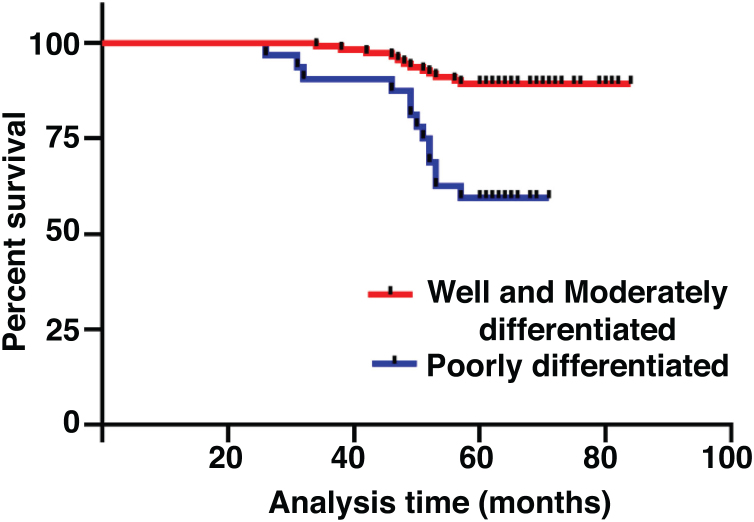
Comparison of 5-year survival rate according to pathological grade (*p* <  0.001).

**Figure 6:**
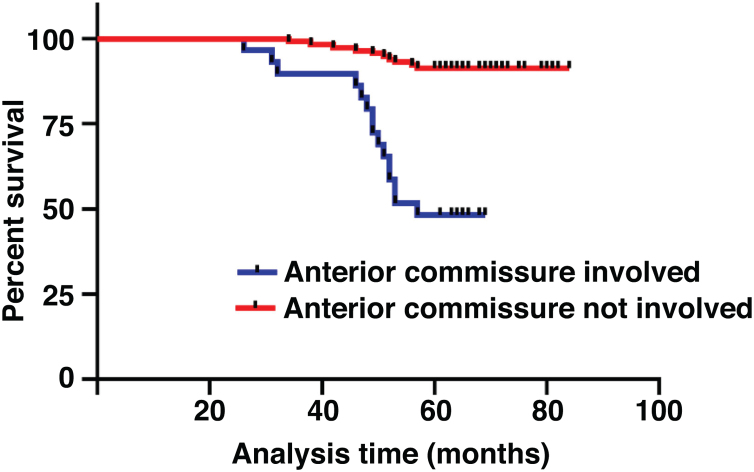
Comparison of 5-year survival rate according to anterior commissure involvement (*p* <  0.001).

The median duration of follow-up was 64 months (range, 26 − 84 months). Table 2 summarized the characteristics of the patients and makes a univariate analysis to identify the factors affecting the 5-year survival rate. The 5-year survival rate of patients less than 60 years old was 86.8%, while that of patients ≥ 60 years old was 71.1% (Fig. 2). There was a significant difference between the two groups (χ2 = 5.245, *p*  = 0.022). In the study, gender has no effect on prognosis (χ2 = 0.001, *p*  = 0.970) (Fig. 3). In univariate analysis, higher T-stage (χ2 = 10.717, *p*  = 0.005) (Fig. 4), poor pathological grading (χ2 = 16.806, *p* < 0.001) (Fig. 5) and anterior commissure involvement (χ2 = 35.193, *p* < 0.001) (Fig. 6) were all significant factors affecting the 5-year survival. In multivariate analysis, age, T-stage, pathological grading, and anterior commissure involvement were still important prognostic factors for early glottic cancer (Table 3).

**Table 2:** Patient characteristics and potential prognostic factors for overall survival.

Prognostic factor	Nº of patients	5-year survival rate (%)	χ^2^	*p*-value
Gender				0.970
Male	132	82.6	0.001	
Female	12	83.3		
Age (years)				0.022
< 60	106	86.8	5.245	
≥ 60	38	71.1		
Treatment				0.987
Open surgery	53	83.0	0.026	
Laser microsurgery	46	82.6		
Radiotherapy	45	82.2		
T-stage				0.005
T_is_	6	100	10.717	
T_1_	77	90.9		
T_2_	61	70.5		
Pathological grading				<0.001
Well and moderately differentiated	112	89.2	16.806	
Poorly differentiated	32	59.4		
Tumor in anterior commissure				<0.001
No	115	91.3	35.193	
Yes	29	48.3

**Table 3:** Prognostic factors for overall survival on multivariate analysis (Cox model).

Prognostic factor			95% CI	
	p-value	HR	Lower	Upper
Age	0.001	0.120	0.035	0.410
Clinical T-stage	0.019	4.758	1.287	17.589
Pathological grading	0.014	3.883	1.323	11.397
Anterior commissure involvement	0.002	18.081	2.983	109.596

## Discussion

Head and neck cancer encompasses a broad spectrum of malignancies, and is responsible for 550,000 new cases and 380,000 deaths worldwide annually.^12^ Histologically, the vast majority (about 90%) are squamous cell carcinomas. The optimal treatment approach for such high-risk patients remains unclear. Glottic cancer patients with clinical manifestations such as hoarseness can be detected in time,^13^, ^14^ and the local lymph node metastasis rate is low,^15^, ^16^ so they have a higher survival rate,^17^ but there are still some patients with intractable disease or tumor recurrence. We retrospectively analyzed the effects of different clinical features and treatments on 5-year survival rates to identify high-risk groups for treatment failure in this subgroup.

The survival rate of cancer patients decreases with the increase of age.^18^ Age is also an important factor in glottic cancer. In our study, patients were divided into ≥ 60 years old and < 60 years old, and it was found that there was a significant difference in 5-year survival rate between the two groups (*p*  = 0.022). This is consistent with the conclusions of other studies.^19^

T stage is well known as a prognostic factor in patients with glottic cancer.^20^ Although it could not be identified as a significant factor in multivariate analysis of some studies,^21^ it still had a significant effect on survival in this study (HR = 4.758, 95% CI 1.287–17.589, *p* = 0.019). Pathological grading is also an important factor affecting the 5-year survival rate (HR = 3.883, 95% CI 1.323–11.397, *p* = 0.014), which is consistent with our clinical experience.

The impact of anterior commissure involvement on local control of glottic cancer has been controversial. Some studies have thought it does not affect the local control rate significantly.^22^, ^23^ Anatomically, the thyroid cartilage lacks protective perichondrium as a potential tumor barrier, while the anterior commissure is directly attached to the thyroid cartilage. This barrier is a weak area from the point of view of tumor dissemination. According to our data, the local failure rate was 18.08 times greater if the anterior commissure was involved. Anterior commissure involvement was also an important prognostic factor in our study (HR = 18.081, 95% CI 2.983–109.596, *p* = 0.002). This is consistent with our previous conjecture. However, the range of 95% CI may be large due to the sample size.

Although some studies have shown that gender is an independent factor affecting the overall survival rate of laryngeal cancer,^24^, ^25^ we have not come to such a conclusion in our study.

In most patients with early glottic cancer, organ preservation is gradually considered to be the best initial treatment. Transoral laser microsurgery and radiotherapy have become important treatments for early glottic tumors. In our study, there was no significant difference in the 5-year survival rate between the two treatments and open surgery. Laser microsurgery requires good exposure of the surgical area to ensure an adequate margin of safety. But for recurrent tumors, this is not a good treatment.^26^, ^27^ It is likely that the direct view of the tumor together with a more extended excision of involved seems to guarantee a better local control after radiotherapy relapse than these achieved by microsurgery. Voice quality is reported to be better after radiotherapy.^22^, ^28^, ^29^ However, when assessed by acoustic analysis and speech aerodynamic studies, the quality of the voice does not return to normal following irradiation.^30^ This is probably due to the tumor itself, or because radiotherapy produces a geometric asymmetry and, henceforth, a loss of elasticity of the vocal folds.^7^ When choosing a treatment plan, we should explain to the patients the advantages and disadvantages of various treatment methods, which are ultimately decided by the patients themselves.

## Conclusion

There was no significant difference in the 5-year survival rate of early glottic cancer treated with radiotherapy, laser microsurgery or open surgery. Multivariate analysis showed that age, T-stage, pathological grading, and anterior commissure involvement were important factors affecting prognosis.

## Conflicts of interest

The authors declare no conflicts of interest.
